# Refugees in the media: Exploring a vicious cycle of frustrated psychological needs, selective exposure, and hostile intergroup attitudes

**DOI:** 10.1002/ejsp.2580

**Published:** 2019-05-17

**Authors:** Adrian Lueders, Mike Prentice, Eva Jonas

**Affiliations:** ^1^ Department of Psychology University of Salzburg Salzburg Austria; ^2^ Department of Psychology Wake Forest University Winston‐Salem North Carolina

**Keywords:** ingroup defense, media, prejudice, psychological needs, refugees, selection bias

## Abstract

Two research objectives underlay the present research. First, we tested how frustrated psychological needs caused by the refugee‐influx influence the endorsement and selection of refugee‐relevant information. Second, we tested how information selection processes contribute to the development of exclusionary attitudes that counteract the integration of refugees into host countries. In a laboratory study (*n* = 181), frustrated psychological needs decreased participants’ endorsement of a refugee‐friendly essay (vs. a control essay). Additionally, frustrated needs led to a biased selection of refugee‐hostile over refugee‐friendly information and such selection biases, in turn, predicted higher levels of ingroup defense and prejudice toward refugees. The findings imply that host societies’ receptiveness to refugees is influenced by the maintenance of basic psychological needs.

## 1 INTRODUCTION

The purpose of the present study is to examine relations among frustrated psychological needs, media consumption, and the development of hostile intergroup attitudes in the context of the so‐called refugee crisis. This is important for at least three reasons. First, attitudes about refugees may play a part in the current spreading of ethnocentric public opinion and right‐wing populism in many European countries. Second, these attitudes have impacted border‐control policies that have led to thousands of refugees drowning while they attempt to illegally cross European boarders. Third, the development of extreme attitudes polarizes European societies and threatens the stability of the European Union as a whole.

### 1.1 The European “refugee crisis”

Ongoing conflicts in the world have forcibly displaced millions of people from their home countries in historically large numbers over the last decade (UNHCR, 2017). States neighboring the conflicts bore the brunt of accommodating incoming asylum claimants, but European countries were also confronted with the consequences of these humanitarian crises. It is reasonable to assume that the successful care for and integration of refugees into host societies relies heavily on the attitudes that community members hold about incoming refugees. However, although many European citizens agree that their countries would benefit from integration in the long run, a significant proportion of people do not believe that governments are managing integration matters adequately and perceive refugees as potential threats (European Commission, 2018; IPSOS, 2017).

### 1.2 Media and the public perception of refugees

Media play a central role in the development and maintenance of public opinions. Content analysis of media coverage from different European countries revealed strong differences in the extent to which media expressed sympathy for refugees and provided contextual information about causes of flight (Berry, Garcia‐Blanco, & Moore, 2015). Prior research showed that threat‐focused depictions of refugees may foster prejudicial attitudes as such depictions stimulate outgroup dehumanization processes and raise intergroup anxiety among host citizens (Esses, Medianu, & Lawson, 2013; Stephan, Renfro, Esses, Stephan, & Martin, 2005). Researchers who explored the effects of the mass harassments that took place during the New Year's Eve celebrations 2015/2016 in Cologne, Germany showed that media widely focused on the perpetrators cultural background to explain the events (Stürmer, Rohmann, Froehlich, & van der Noll, 2018). Additionally, the authors demonstrated that the endorsement of such essentialist explanations amplified the effect of symbolic threat on restrictive policy support and approval of right‐wing aggression (Studies 2 and 3).

Despite the possibility that negative media portrayals can bias public opinion against refugees, there is evidence indicating that more favorable and empathic depictions of refugees may counteract such detrimental effects (Florack, Piontkowski, Rohmann, Balzer, & Perzig, 2003; Stephan et al., 2005). However, intrapersonal factors may undermine the potential benefits of positive portrayals of refugees. For instance, citizens with refugee‐hostile attitudes may denigrate refugee‐friendly coverage to defend their existing beliefs (Edwards & Smith, 1996; Vallone, Ross, & Lepper, 1985). Citizens may also craft their information diets in ways that enable them to avoid attitude‐challenging information in the first place. People may therefore end up in “filter bubble” environments and consolidate refugee‐hostile opinions due to selective exposure to attitude‐reinforcing information (Sunstein, 2018). To better understand how media correspond to the development and maintenance of exclusionary convictions, researchers must therefore consider not only bottom‐up processes that foster the endorsement and selection of refugee‐hostile media, but also potential top‐down effects of such media diets on intergroup perceptions.

### 1.3 Psychological needs and refugee perception

Research suggest that host society members who perceive refugees as a threat to psychological needs (i.e., for certainty, belonging, control, meaning, and self‐esteem) are more prone to embrace exclusionary attitudes as well as to craft media diets that bolster these opinions. Needs can be understood as motivational forces that push individuals toward a desired state of optimal psychological functioning (e.g., Deci & Ryan, 2000). For instance, people want to feel socially embedded and hold a positive representation of themselves (Leary & Baumeister, 2000; Steele, 1988). People also seek to exert control over their environment and perceive their surroundings as comprehensible and predictable (Kruglanski & Orehek, 2011; Skinner, 1996). Finally, people seek to experience their lives as meaningful, which includes feelings of personal significance and purposeful acting (Martela & Steger, 2016).

While need satisfaction positively affects psychological well‐being, need frustration is perceived as aversive and stimulates psychological mechanisms to restore thwarted needs (Jonas et al., 2014). Researchers noted that in response to external events that potentially threaten psychological needs, people may gravitate toward exclusionary ideologies (Cichocka, 2016; Hogg, Kruglanski, & van den Bos, 2013; Jost, 2017). Congruently, polls from Germany revealed that supporters of the populist right‐wing party *Alternative for Germany* (AFD) feel more uncertain about the future and less capable of altering political decisions as compared to supporters of other parties (Elmer & Meiritz, 2016; Peterson, 2016). An explanation of these effects lies in the function of groups in conferring individual needs with abstract benefits that derive from social identities (Abrams & Hogg, 2006; Correll & Park, 2005; Greenaway, Cruwys, Haslam, & Jetten, 2016; Jetten, Haslam, & Alexander, 2012). Defending one's social identity may thus be a functional tool to regulate need frustration on the personal level. On the social level, however, such responses may carry a range of undesirable outcomes. Accordingly, research provided evidence that need frustration (e.g., due to self‐evaluation threats, control deprivation, self‐uncertainty, ostracism, or significance loss) fosters ingroup defense and outgroup derogation (Cichocka, 2016; Fritsche et al., 2013; Hogg, Meehan, & Farquharson, 2010; Jasko, LaFree, & Kruglanski, 2017; Schaafsma & Williams, 2012).

Although such studies help scholars understand the motivational underpinnings of intergroup hostility, many researchers have treated need frustration rather as an abstract phenomenon than as an outcome of particular social events. Exploring need frustration in particular social contexts, however, may improve possibilities to infer targeted practical implications. In the present research, we focus on need frustration in the context of the European refugee influx to simultaneously examine antecedents and outcomes of refugee‐hostile media consumption. We refer to thwarted perceptions of control as the feeling that someone must accept decisions without being able to alter the events. Additionally, we refer to frustrated feelings of belonging if a person feels socially isolated. We conceptualize thwarted perceptions of self‐esteem as feelings of disregard combined with unfavorable self‐evaluations. Finally, we expect needs for meaning and certainty to be frustrated to the extent that someone feels unable to understand and meaningfully respond to the refugee situation.

### 1.4 Need frustration and media consumption

Research suggests that apart from fostering negative attitudes about refugees, need frustration simultaneously increases the motivation to seek attitude‐supportive media coverage. A meta‐analysis that examined effects of selective exposure to attitude‐reinforcing information revealed that people hold a moderate preference to favor attitude‐consistent over attitude‐challenging information (Hart et al., 2009). Further research indicated that this tendency increases if psychological needs are at stake. For example, researchers found that temporal motivations to reach closure increased selection biases among participants with strong chronical needs for certainty (i.e., high need for closure; Hart, Adams, Burton, Shreves, & Hamilton, 2012). Moreover, after reminding participants of their mortality (which can be understood as a global need threat), participants performed enhanced selection biases for worldview‐confirming information in comparison to a control group (Jonas, Greenberg, & Frey, 2003; Lavine, Lodge, & Freitas, 2005). Additionally, Fischer et al. (2011) observed pro‐attitudinal selection biases after letting participants reflect upon threatening socio‐political topics (e.g. terrorism, financial crisis).

Using Festinger's theoretical framework, researchers explained selective exposure effects through people's motivation to avoid dissonance by maintaining consonant cognitions (Festinger, 1957; Frey & Stahlberg, 1986). On a broader level, however, seeking attitude‐consistent information may also help people maintain perceptions of certainty and meaning, especially when the context of information refers to their general assumptions about the world (Jonas et al., 2003). Moreover, research showed that controversial (e.g., political) topics can trigger social categorization processes that activate partisan identities (Knobloch‐Westerwick & Meng, 2011). Seeking partisan information may then enhance feelings of belonging and let groups appear as more agentic (Jost, van der Linden, Panagopoulos, & Hardin, 2018; Stern, West, Jost, & Rule, 2014). Conversely, derogating counter partisan information defends the superior image of the ingroup through which individuals can regulate their need for self‐esteem (Hartmann & Tanis, 2013).

To meet the complexity of field settings in which people are facing myriads of information at the same time, researchers applied latent space models to analyze potential selective exposure effects among social media users. Following the theoretical claim according to which conservatives are more prone to need frustration than liberals (Jost, Glaser, Kruglanski, & Sulloway, 2003), researchers explored the retweet behavior of 3.8 million twitter users based on their ideological preferences (Barberá, Jost, Nagler, Tucker, & Bonneau, 2015). As expected, results showed that conservatives hold a stronger tendency than liberals to exchange attitude‐reinforcing information with like‐minded others. Researchers showed that continuous exposure to attitude‐reinforcing information may increase both, attitude and partisan strength (Knobloch‐Westerwick, 2012). Hence, when applying such findings to the topic of refugees, it seems likely that a biased consumption of refugee‐hostile information would increase xenophobia among citizens who are already suspicious of refugees.

**Table 1:** Means and standard deviations (in parentheses) for continuous variables in the different conditions

	Refugee‐friendly essay (*n* = 60)	Refugee‐hostile essay (*n* = 61)	Neutral essay (*n* = 60)
Need frustration	3.21 (0.66)	3.16 (0.68)	2.92 (0.61)
Essay endorsement	4.25 (1.36)	2.36 (1.14)	3.50 (1.25)
Selective exposure	0.02 (1.13)	0.36 (0.71)	0.35 (0.94)
Ingroup defense	3.40 (0.82)	3.35 (0.77)	3.41 (0.77)
Prejudice	2.31 (1.03)	2.23 (0.84)	2.23 (0.87)

### 1.5 Examining a vicious cycle of frustrated psychological needs, selective exposure, and hostile intergroup attitudes

**Figure 1:**
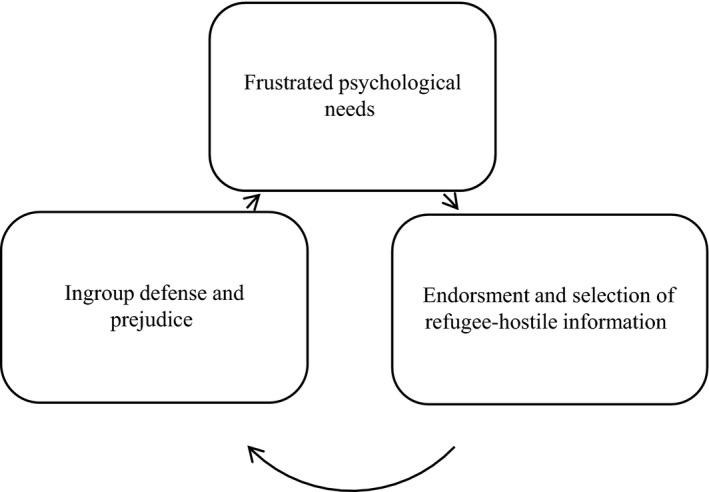
Conceptual representation of the proposed vicious cycle: Frustrated psychological needs increase the endorsement and selection of refugee‐hostile information. Refugee‐hostile information diets in turn foster ingroup defense and prejudice toward refugees

In the present research, we propose a vicious cycle in which frustrated needs caused by the influx of refugees into Europe increase the endorsement of refugee‐hostile media coverage (H1a) and decrease the endorsement of refugee‐friendly media coverage (H1b), respectively. Moreover, we expect frustrated needs to enhance the motivation to seek attitude‐reinforcing (i.e., refugee‐hostile) over attitude‐challenging (i.e., refugee‐friendly) information (H2). Finally, we expect selective exposure to refugee‐hostile information to mediate the effects of need frustration on ingroup defense (H3) and prejudice toward refugees (H4) (Figure 1).

## 2 METHOD

### 2.1 Participants

We aimed to collect 150–200 participants in the available time frame and finally generated a sample of *n* = 181. Participants were mainly undergraduate psychology students from the University of Salzburg who participated in exchange for course credit. We analyzed the data after the full sample had been collected and collected no further data. Sensitivity power analysis revealed that our sample enabled us to detect small single regression effects of *f *=* *0.19, and medium interaction effects of *f *=* *0.26 (Cohen, 1988; Faul, Erdfelder, Lang, & Buchner, 2007). The sample's age ranged from 17 to 35 years (*x*_age_ = 21.99; *SD *=* *2.94). Most participants were German (72.4%) or Austrian (24.9%) citizens (Others = 2.8%). Gender distribution revealed a small majority of female participants (56.9%).

### 2.2 Procedure and material

Participants were informed that the study involved personality and online media consumption. Data was collected in a university‐based laboratory where up to four participants were simultaneously tested in isolated cubicles. Materials were presented on a computer screen.

We describe the material in the same order as it appeared in the survey. Continuous items were rated on a 6‐point scale (1 = *strongly disagree* to 6 = *strongly agree*) and collapsed into composite scores.

#### 2.2.1 Psychological need frustration

Before responding to the items, we asked participants to think about their thoughts and feelings raised by the current refugee situation. The scale was based on a measure to assess need threats caused by ostracism (Zadro, Williams, & Richardson, 2004). The original scale assessed personal needs for control, self‐esteem, belonging, and meaning. We added three items to assess certainty, due to the particular relevance of (un)certainty to the refugee situation (c.f. Esses et al., 2013). Previous research has averaged all the items into a general indicator of need frustration (Greenaway et al., 2016; Zadro et al., 2004). This treatment of the measure has since been supported by psychometric evaluations (Gerber, Chang, & Reimel, 2017). Factor analysis of the current data recommended a bifactor solution with a general need factor (CFI = 0.80, RMSEA = 0.105) over an oblique, multi‐factorial one (CFI = 50, RMSEA = 0.150). We therefore focus further analysis on global need frustration. All items loaded significantly on the global factor in the factor analysis, and a composite measure indicating satisfactory scale reliability (*α* = 0.79; *x *=* *3.09, *SD *=* *0.66).

The whole measure contained 15 items, three items for each particular need (e.g., control: *I have to accept many things that I cannot alter myself*; belonging: *I feel isolated by others*; self‐esteem: *I feel valued as a person*; certainty: *I have the feeling that I don't fully understand the situation*; meaningful existence: *Sometimes, I feel useless*).

#### 2.2.2 Endorsement of media coverage

Next, we examined the effect of need frustration on the endorsement of refugee‐friendly and refugee‐hostile information in comparison to a control condition. Participants were randomly assigned to one of three essay conditions. A refugee‐friendly essay highlighted economic and demographic opportunities associated with refugees. A refugee‐hostile essay depicted refugees as physical and cultural threats. A control essay contained information about immigration laws in Canada. Essays were created based on available mass media in Germany and Austria. All essays were discussed by at least two researchers to ensure their comparability with real media. To ensure that participants read each essay carefully, we informed them that they would later receive further questions about the text. Indicative for participants’ perception of refugees (i.e., as benefits or threats), participants expressed their endorsement of the essays with a single item (*To what extent does the article reflect your personal opinion?*;* x *=* *3.36, *SD *=* *1.47). Higher item‐scores indicated higher levels of endorsement.

**Table 2:** Standardized regression weights and squared multiple correlations

	Estimate	95% CI
*Standardized regression weights*		
Need frustration → Selective exposure	0.34^c^	[0.17, 0.49]
Essay_refugee‐friendly_ → Essay endorsement	0.27^c^	[0.12, 0.40]
Essay_refugee‐friendly_ × Need frustration → Essay endorsement	−0.21^a^	[−0.44, −0.01]
Need frustration → Essay endorsement	0.03	[−0.22, 0.28]
Essay_refugee‐hostile_ × Need frustration → Essay endorsement	0.06	[−0.11, 0.25]
Essay_refugee‐hostile_ → Essay endorsement	−0.38^c^	[−0.52, −0.24]
Selective exposure → Ingroup defense	0.21^b^	[0.04, 0.36]
Selective exposure → Prejudice	0.26^c^	[0.14, 0.38]
Need frustration → Ingroup defense	−0.06	[−0.20, 0.11]
Need frustration → Prejudice	0.38^c^	[0.25, 0.51]
*Squared multiple correlations*		
Selective exposure	0.12^c^	[0.03, 0.24]
Prejudice	0.28^b^	[0.16, 0.40]
Ingroup defense	0.04^b^	[0.01, 0.10]
Essay endorsement	0.32^b^	[0.20, 0.42]

a *p* < 0.05,

b *p* < 0.01,

c *p* < 0.001.

#### 2.2.3 Selective exposure

Next, we presented participants with four different newspaper headlines (in random order) to examine how frustrated psychological needs influence the selection of attitude‐reinforcing and attitude‐challenging information. Two headlines announced refugee‐friendly articles while the other two announced refugee‐hostile articles (friendly: *Huge knowledge resource: Contact with refugees and their problems holds chance for society to develop more complex worldview*;* Economic forecast: Migrants improve growth prospects. Economists sure that Europe can benefit from the influx of refugees. Not only would population grow, so would economy*; hostile: *ISIS fighters among refugees: Large influx of refugees thwarts security measures*;* Refugee issue tearing Europe apart: Situation along the German‐Austrian border is getting worse. CSU leader Seehofer suggests Bavaria prepared to defend itself)*. After each headline, participants could decide to read the full article or skip to the next headline**.** In accordance with prior research (e.g., Fischer et al., 2011), we calculated selective exposure scores by subtracting the number of selected attitude‐reinforcing articles from the number of attitude‐challenging articles (*x *=* *0.24; *SD *=* *0.95).

#### 2.2.4 Ingroup defense and prejudice

Finally, we tested the effect of selective exposure on prejudice and ingroup defense. The ingroup defense measure contained nine items (Fritsche, Jonas, & Fankhänel, 2008) and assessed aspects such as ingroup identification (e.g., *I strongly identify with my culture*) and perceived ingroup homogeneity (e.g., *The people in my culture from a homogeneous group*;* α* = 0.78; *x *=* *3.39, *SD *=* *0.78).

The prejudice measures contained of six items that assessed negative outgroup beliefs (e.g., *I'm suspicious toward incoming refugees)* and perceived intergroup anxiety *(e.g., I feel threatened by incoming refugees*;* α* = 0.90; *x *=* *2.25, *SD *=* *0.91).

Finally, we assessed sociodemographic data and carefully debriefed each participant.

## 3 RESULTS

### 3.1 Analytical strategy

Parameters were estimated via maximum likelihood method. Indirect effects were calculated via user‐defined estimands with 5,000 bootstrap samples and 95% confidence intervals in AMOS 22. The full model is described in Figure 3. Table 1 provides means and standard deviations of the continuous variables.

We expected frustrated psychological needs to increase the endorsement of refugee‐hostile coverage (H1a) and to decrease the endorsement of refugee‐friendly coverage (H1b), respectively. We calculated two dummy variables to compare the refugee‐hostile and the refugee‐friendly essay with the control essay. For the interaction terms, we multiplied each dummy variable with the mean‐centered variable “needs frustration”.

The results revealed a significant negative main effect of the refugee‐hostile essay on endorsement, indicating that participants showed lower endorsement of the refugee‐hostile essay relative to the control essay, *b *=* *−1.17, *SE *=* *0.225, *p *<* *0.001; 95% CI [−1.63, −0.75]. A non‐significant *essay*_*hostile*_ *× needs* interaction term (*p *=* *0.532) indicated that psychological needs had no substantial effect on the endorsement of the refugee‐hostile essay. A significant positive main effect indicated that participants endorsed the refugee‐friendly essay more than the control essay, *b *=* *0.825, *SE *=* *0.228, *p *<* *0.001; 95% CI [0.37, 1.31]. This effect was qualified by a significant *essay*_*friendly*_ *× needs* interaction, *b *=* *−0.819, *SE *=* *0.353, *p *=* *0.020; 95% CI [−1.58, −0.03]. As expected, simple slopes analysis revealed that frustrated needs significantly reduced the endorsement of the refugee‐friendly essay, *b *=* *−0.75, *t* (175) = −3.09, *SE *=* *0.24, *p *=* *0.002, 95% CI [−1.23, −0.27]. The simple effect of needs on the control essay was not significant (*p *=* *0.274).

**Figure 2:**
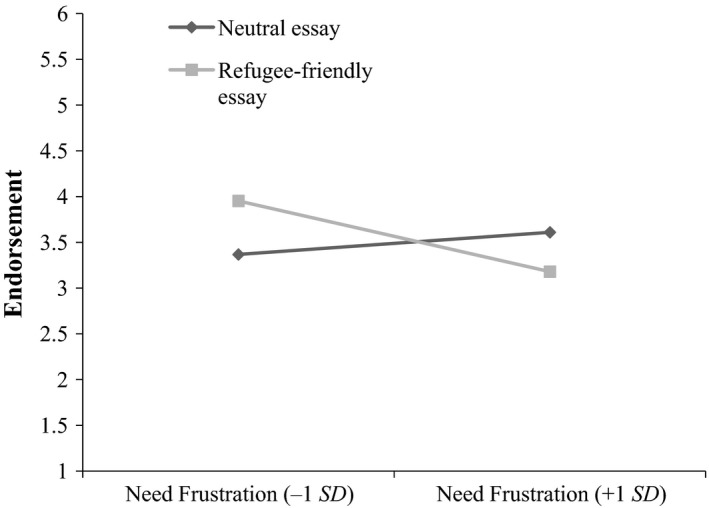
Frustrated psychological needs reduce the endorsement of a refugee‐friendly essay in comparison to a neutral essay (H1b)

The results reject Hypothesis 1a, as frustrated psychological needs did not significantly increase participants’ endorsement of a refugee‐hostile essay relatively to a control essay. In line with Hypothesis 1b, frustrated psychological needs reduced the endorsement of media coverage that depicts refugees as an opportunity (Figure 2).

Next, we tested our prediction, that need frustration would predict selective exposure to refugee‐hostile information (H2) and through this ingroup defense (H3) and prejudice (H4).

In accordance with Hypothesis 2, results showed that frustrated needs predicted enhanced selection biases for refugee‐hostile information, *b *=* *0.494, *SE = *0.101, *p *<* *0.001; 95% CI [0.24, 0.73]. Moreover, selection biases positively predicted ingroup defense, *b *=* *0.17, *SE = *0.064, *p *=* *0.010; 95% CI [0.03, 0.29] and prejudice, *b *=* *0.25, *SE = *0.065, *p *<* *0.001; 95% CI [0.13, 0.38].

**Figure 3:**
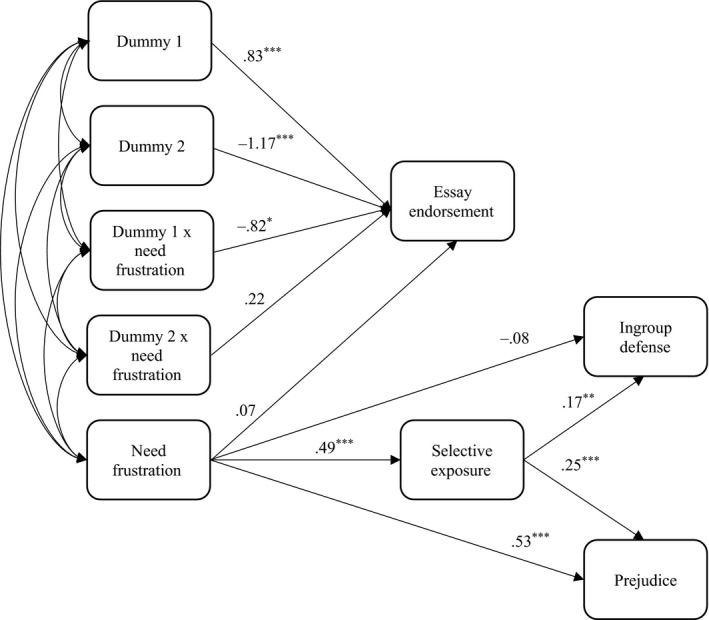
Structural Equation Model, *x* ^2^ */df* = 1.378; CFI = 0.996; RMSEA = 0.046. *Note:* Dummy 1: Refugee‐friendly essay = 1; Refugee‐hostile essay = 0; Control essay = 0. Dummy 2: Refugee‐friendly essay = 0; Refugee‐hostile essay = 1; Control essay = 0. The model shows the unstandardized regression coefficients. For standardized coefficients see Table 2. *p < 0.05; **p < 0.01; ***p < 0.001

Analysis of indirect effects supported our proposed path model. In accordance with Hypothesis 3, a significant indirect effect indicated that need frustration predicted higher levels of ingroup defense due to selective exposure to refugee‐hostile information, *b *=* *0.09, *p *=* *0.006, 95% CI [0.18, 0.02]. In accordance with Hypothesis 4, a significant indirect effect indicated that need frustration also increased the reported level of prejudice toward refugees via selective exposure to refugee‐hostile information, *b *=* *0.124, *p *=* *0.001, 95% CI [0.22, 0.06] (Figure 3).

## 4 DISCUSSION

The first goal of the present research was to examine how frustrated psychological needs deriving from the overall refugee situation relate to the endorsement and selection of refugee‐friendly and refugee‐hostile media. The second goal was to test whether a biased consumption of refugee‐hostile information would predict increased levels of ingroup defense and prejudice toward refugees.

Although need frustration did not predict participants’ endorsement of a refugee‐hostile essay (H1a), need frustration negatively predicted endorsement of a refugee‐friendly essay (H1b). A data driven explanation for the rejection of Hypothesis 1a could lie in the refugee‐friendly tendency of our sample. A theoretical explanation could be that need frustration had a stronger effect on participants’ motivation to disapprove an attitude‐challenging essay compared to participants' motivation to endorse an attitude‐reinforcing essay. Accordingly, researchers found that people spend more efforts on scrutinizing attitude‐incongruent versus attitude‐congruent information to defend existing beliefs (Edwards & Smith, 1996). Moreover, research on “hostile media effects” suggests that people may perceive attitude‐challenging media as unfairly biased against their own viewpoints (Perloff, 2015). In two studies, negative perceptions of refugees predicted biased media perceptions, suggesting that need deprivation may indeed be involved in such effects (Arlt, Dalmus, & Metag, 2018; Arlt & Wolling, 2016). However, future studies containing samples that better represent the general population are needed to examine these possibilities.

In accordance with Hypothesis 2, frustrated needs predicted stronger selection biases for information that validated participants’ negative perceptions of refugees. This finding provides important theoretical and practical implications. On a theoretical level, it resonates with field‐studies that found selective exposure effects being moderated by ideological partisanship (e.g., Barberá et al., 2015; Boutyline & Willer, 2017). Congruent with the claim that ideological asymmetry effects derive from differences in need‐preservation motives, we provided evidence that need frustration indeed relates to stronger attitude‐reinforcing information seeking. On a practical level, the results indicate that discriminatory and fear‐generating media benefits to a certain degree from enhanced levels of need frustration associated with the “refugee crisis”.

In a last step, we examined the effects of refugee‐hostile media diets on group‐related attitudes. As expected, selective exposure to refugee‐hostile information predicted increased levels of ingroup defense and prejudice. Moreover, and in line with our predicted path model, two significant indirect effects indicated that selective exposure to refugee‐hostile information mediated the effects of need frustration on ingroup defense (H3) and prejudice (H4).

To the best of our knowledge, the present findings are the first that (a) predict selective exposure effects with increased levels of need frustration deriving from a particular social context and (b) simultaneously investigate how motivated media selection may contribute to hostile intergroup attitudes. However, although we based our model on a theoretical fundament the present results do not allow us to draw causal conclusions. To address this limitation, future experiments could manipulate psychological needs to examine causal effects of need frustration on media behavior and intergroup perceptions (Spencer, Zanna, & Fong, 2005).

Future research might also include factors that were not part of the present research yet might have influenced the observed effects. For example, researchers might focus on certain emotions elicited by need frustrations and examine their effects on media selection and group‐based cognitions. In fact, an ironic consequence of selectively seeking refugee‐hostile coverage is that people isolate themselves from information that could alleviate threat perceptions by putting refugees into a more favorable light. This result seems to be consistent with prior findings that have challenged the idea according to which media selection is mainly governed by hedonic mood optimization (Zillmann, 2000). However, following Knobloch's (2003) claim that people seek to adjust their mood to the requirements of a particular context one might argue that seeking anxiety‐evoking information about refugees may be more adequate than seeking anxiety‐relieving information for citizens who advocate for policies of closure (c.f. Jonas, Graupmann, & Frey, 2006).

Additionally, researchers might also investigate how different forms and components of media may foster radicalization. Especially in the context of social media consumption, such research could include social interaction processes, such as (aggressive) partisan rhetoric and digital indicators of social endorsement (c.f. Soral, Bilewicz, & Winiewski, 2018; Messing & Westwood, 2014).

Finally, researchers might seek strategies to explore contextual and intrapersonal factors that may help explain why some people report higher levels of need frustration than others. Unfavorable social comparisons, perceptions of procedural injustice, or status threats might thereby be promising starting path.

## 5 IMPLICATIONS FOR THE INTEGRATION OF REFUGEES

These present findings indicate that the general appraisal of the current situation of refugees in Europe is determined by the level of need frustration it causes among host society members. Media and politicians may fuel these effects by depicting refugees in an essentialist and threat‐focused manner (Esses et al., 2013). However, the findings also indicate that bolstering psychological needs may serve as a strategy to counteract nationalism and xenophobia in response to social challenges. Accordingly, findings from experimental social psychology showed that bolstering psychological needs may mitigate exclusionary threat‐responses (Aydin, Krueger, Frey, Kastenmüller, & Fischer, 2014; Greenaway, Louis, Hornsey, & Jones, 2014). Communicating refugee matters in a way that preserves psychological needs could therefore help in maintaining or even improving the receptiveness of host society members and therefore foster the integration of refugees.

## CONFLICT OF INTEREST

The authors confirm that there are no known conflicts of interest associated with this publication.

## ETHICAL STATEMENT

The authors confirm that the manuscript adheres to ethical guidelines specified in the APA Code of Conduct as well as authors’ national ethics guidelines.

## TRANSPARENCY STATEMENT

Electronic copies of data, codes, and materials have been stored at an institutional data repository. as well as on an US‐based OSF server (https://osf.io/5hqke/). Supplementary materials can be retrieved via the OSF Link and as online supplementaries.
