# Supplementary material for: Refugees in the media: Exploring a vicious cycle of frustrated psychological needs, selective exposure, and hostile intergroup attitudes
Source: Eur J Soc Psychol. 2019 May 17;49(7):1471–9. doi: 10.1002/ejsp.2580 (PMC6919923; doi:10.1002/ejsp.2580)
Supplement: Supplementary file 1 [file EJSP-49-1471-s001.docx]

**Supplementary Material**

**Appendix A: Additional Measures**

Need for closure (Schlink, & Walther, 2007).

Justice Sensitivity (Beierlein et al. 2012).

Rosenberg Self-Esteem (Roth, Decker, Herzberg, & Brähler, 2008).

Implicit Self-Esteem (Greenwald & Farnham, 2001).

Manikin Task: Ingroup vs. Outgroup Approach/Avoidance (e.g. De Houwer, Crombez, Baeyens, & Hermans, 2001).

**References**

Beierlein, C., Baumert, A., Schmitt, M., Kemper, C. J., Kovaleva, A., & Rammstedt, B. (2012). Kurzskalen zur Messung der Ungerechtigkeitssensibilität: Die Ungerechtigkeitssensibiliät-Skalen-8 (USS-8). GESIS Working Papers, 2012|21.

De Houwer, J., Crombez, G., Baeyens, F. & Hermans, D. (2001). On the generality of the affective Simon effect. *Cognition and Emotion, 15,* 189–206.

Greenwald, A. G., & Farnham, S. D. (2000). Using the implicit association test to measure self-esteem and self-concept. *Journal of Personality and Social Psychology, 79*, 1022-38.

Roth, M., Decker, O., Herzberg, P. Y., & Brähler, E. (2008). Dimensionality and norms of the Rosenberg Self-Esteem Scale in a German general population sample. *European Journal of Psychological Assessment*, *24*, 190-197.

Schlink, S., & Walther, E. (2007). Kurz und gut: Eine deutsche Kurzskala zur Erfassung des Bedürfnisses nach kognitiver Geschlossenheit. *Zeitschrift für Sozialpsychologie*, *38*, 153-161.

**Appendix B: Material**

**Psychological Need Frustration (c.f. Zadro, Williams, & Richardson, *JESP*, 2004)**

*Einleitung: Welche Gefühle und Gedanken ruft die aktuelle Flüchtlingssituation bei Ihnen hervor?*

(Introduction: What thoughts and feelings do you have when you think about the current situation of refugees?)

*Ich habe das Gefühl, die Situation im Ganzen nicht richtig zu durchschauen.*

(I have the feeling that I don´t fully understand the situation.)

*Ich habe das Gefühl einen wertvollen Beitrag zu leisten.*

(I have the feeling that I can make a valuable contribution.)

*Ich fühle mich von anderen isoliert.*

(I feel isolated by others.)

*Ich muss mich mit vielem abfinden, auf das ich keinen Einfluss habe.*

(I have to accept many things that I cannot alter myself.)

*Ich trete den Herausforderungen selbstbewusst entgegen.*

(I confidently face the upcoming challenges.)

*Ich fühle mich durch die Flüchtlingssituation verunsichert.*

(I feel uncertain due to the situation of refugees.)

*Ich habe das Gefühl, den Entwicklungen ausgeliefert zu sein.*

(I have the feelings that I have no control over the events.)

*Ich habe manchmal das Gefühl, nicht wirklich dazuzugehören.*

(Sometimes, I´m lacking a sense of belonging.)

*Ich fühle mich manchmal nutzlos.*

(Sometimes, I feel useless.)

*Ich fühle mich gut in eine Gruppe integriert.*

(I feel well integrated into a group.)

*Ich habe das Gefühl, dass ich persönlich nur wenig bewirken kann.*

(I have the feeling that I alone have little influence.)

*Ich bin mir manchmal im Unklaren darüber, was genau von mir erwartet wird.*

(Sometimes I´m not sure what exactly is expected from me.)

*Ich habe das Gefühl, einen sinnvollen Beitrag zu leisten.*

(I have the feeling that I can make a meaningful contribution.)

*Ich habe eine positive Einstellung zu mir selbst.*

(I have a positive attitude towards myself.)

*Ich fühle mich als Person wertgeschätzt.*

(I feel valued as a person.)

**Ingroup Defense (Fritsche, Jonas, & Fankhänel, *JPSP*, 2008)**

*Die Mitglieder meiner Kultur bilden eine homogene Einheit.*

(The people in my nation form a homogeneous group)

*Die Mitglieder meiner Kultur sind sich untereinander ziemlich ähnlich.*

(The people in my nation are relatively similar to one another)

*Meine Kultur bildet ein stimmiges Ganzes.*

(My nation forms a coherent whole)

*Die Mitglieder meiner Kultur verfolgen gemeinsame Ziele und Werte.*

(Germans share common goals and a common fate)

*Ich identifiziere mich mit meiner Herkunft.*

(I identify with my nation)

*Meiner Kultur anzugehören hat nichts mit meiner persönlichen Identität zu tun.*

(Belonging to my nation has nothing to do with my identity)

*Mit meiner Kultur verbindet mich eigentlich überhaupt nichts.*

(Nothing connects me with my nation)

*In meiner Kultur entdecke ich mich selbst.*

(In my nation I recognize myself)

*Die Menschen in meiner Kultur haben fast alle ein ähnliches Gemüt.*

(Germans share a common nature)

**Prejudice towards refugees**

*Ich stehe den hier ankommenden Flüchtlingen eher misstrauisch gegenüber.*

*(I´m suspicious towards incoming refugees.)*

*Ich bin den hier ankommenden Flüchtlingen gegenüber aufgeschlossen.*

*(I´m open to incoming refugees.)*

*Insgesamt bin ich eher skeptisch, was die hier ankommenden Flüchtlinge angeht.*

*(Overall, I´m sceptical about incoming refugees.)*

*Ich fühle mich durch die hier ankommenden Flüchtlinge bedroht.*

*(I feel threatened by incoming refugees.)*

*Ich sehe in der derzeitigen Flüchtlingssituation eine Herausforderung, die wir meistern können.*

*(I understand the current situation of refugees as a challenge that we can handle.)*

*Ich habe gegenüber den hier ankommenden Flüchtlingen eine wohlwollende und einladende Haltung.*

*(I have a benevolent and welcoming attitude towards incoming refugees.)*

**Headlines**

Refugee-friendly:

*Eine riesige Wissensressource: Die Konfrontation mit Flüchtlingen und ihren Problemen birgt die Chance, als Gesellschaft ein komplexeres Weltbild zu entwickeln.*

*(Huge knowledge resource: Contact with refugees and their problems holds chance for society to develop more complex worldview.)*

*Konjunktur Prognose: Migranten verbessern Wachstumsaussichten. Ökonomen sind sich sicher: Europa kann vom Zuzug der Flüchtlinge profitieren. Denn nicht nur die Bevölkerung könnte wachsen, sondern auch die Wirtschaft.*

*(Economic forecast: Migrants improve growth prospects. Economists sure that Europe can benefit from the influx of refugees. Not only would population grow, so would economy.)*

Refugee-hostile:

ISIS Kämpfer unter den Flüchtlingen: Der große Ansturm von Flüchtlingen hat alle Sicherheitssysteme außer Kraft gesetzt.

(*ISIS fighters among refugees: Large influx of refugees thwarts security measures.)*

Das Flüchtlingsthema zerreißt Europa: Die Lage an der deutsch-österreichischen Grenze spitzt sich zu. CSU-Chef Seehofer droht mit einer „Notwehr“ Bayerns.

(*Refugee issue tearing Europe apart: Situation along the German-Austrian border is getting worse. CSU leader Seehofer suggests Bavaria prepared to defend itself.)*
